# Optimal Timing of the Neutrophil-to-Lymphocyte Ratio and Platelet-to-Lymphocyte Ratio as Early Predictors of Neurological Outcomes in Postcardiac Arrest Patients

**DOI:** 10.3390/life14111421

**Published:** 2024-11-04

**Authors:** Dongju Kim, Hanna Park, Sang-Min Kim, Won Young Kim

**Affiliations:** Department of Emergency Medicine, Asan Medical Center, University of Ulsan College of Medicine, Seoul 05505, Republic of Korea; skdk39@naver.com (D.K.); park.hanna15@gmail.com (H.P.); swdarkhorse@gmail.com (S.-M.K.)

**Keywords:** cardiac arrest, neuroprognostication, neutrophil-to-lymphocyte ratio, platelet-to-lymphocyte ratio

## Abstract

The neutrophil-to-lymphocyte ratio (NLR) and platelet-to-lymphocyte ratio (PLR) have been recognized as predictors of various critical illnesses. Our study aimed to investigate whether the NLR and PLR measured at different timepoints could predict poor neurological outcomes at 6 months. This observational retrospective cohort study included adults who had experienced out-of-hospital cardiac arrest (OHCA) and received targeted temperature management between November 2015 and December 2020. Patients with an active infection, as confirmed by an initial blood culture, were excluded. Multivariate logistic regression models were used to determine the association between the NLR and PLR at 0, 24, and 48 h after return of spontaneous circulation and poor neurological outcomes, defined as a Cerebral Performance Category score of ≥3 at 6 months. The NLR at 24 h, but not the NLR or PLR at other timepoints, was significantly associated with poor neurological outcomes (odds ratio: 1.05; 95% CI: 1.01–1.09; *p* = 0.018). The NLR at 24 h showed moderate accuracy in predicting poor neurological outcomes, with an AUC of 0.619. A cutoff value of 9.0 achieved 72.5% sensitivity and 47.7% specificity. The NLR measured at 24 h after ROCS could be used for early neuroprognostication given its low cost and widespread availability.

## 1. Introduction

Immediately after return of spontaneous circulation (ROSC), patients with out-of-hospital cardiac arrest (OHCA) may experience postcardiac arrest syndrome, which is characterized by postanoxic brain injury, cardiovascular impairment, and a systemic inflammatory response involving features similar to those observed during sepsis [1,2]. The magnitude of the inflammatory response resulting from whole-body ischemic–reperfusion injury has been strongly associated with the severity of cardiac dysfunction and mortality [3]. Targeted temperature management (TTM) has been implemented to suppress systemic inflammation, modulate circulating leukocytes, and promote platelet activation [4].

A complete blood count (CBC), a blood test used to evaluate overall health status, is necessary for OHCA patients receiving TTM. Recently, several new white blood cell-based inflammatory indices, such as the neutrophil-to-lymphocyte ratio (NLR), platelet-to-lymphocyte ratio (PLR), red cell distribution width, and mean platelet volume, have been introduced [5,6]. Among the mentioned indices, the NLR and PLR have been described as rapid, inexpensive, and easily reproducible markers of systemic inflammation. Several stress-inducing events, such as sepsis and cardiac arrest, may cause accelerated lymphocyte apoptosis and delayed neutrophil apoptosis [7]. As such, a higher NLR has been associated with lower survival rates in various diseases, including cardiac arrest [8,9,10]. Nevertheless, limited evidence has been available regarding the relationship between the NLR and PLR in postcardiac arrest survivors due to the small cohort sizes, the focus on mortality as the primary outcome, or failure to exclude bacterial infections [11,12,13,14,15]. Additionally, each value represents a different mechanism in the inflammatory process and follows a distinct time course throughout post-cardiac arrest care [16,17].

We hypothesized that the NLR and PLR, hematological markers of inflammation routinely measured from CBC panels, could be potentially useful for the early prognostication of patients with OHCA after TTM. Hence, the current study aimed to determine whether the NLR and PLR measured at difference timepoints could predict neurological outcomes among survivors of OHCA undergoing TTM.

## 2. Materials and Methods

### 2.1. Study Design and Population

This retrospective cohort study analyzed data obtained from a prospectively collected registry of OHCA patients who underwent TTM in seven tertiary referral centers across South Korea from November 2015 to December 2020. The inclusion criteria for the registry comprised comatose adults (aged > 18 years) with nontraumatic OHCA who received TTM. The exclusion criteria included active intracranial bleeding or acute ischemic stroke, limitations in therapy, a do-not-attempt resuscitation order, and a Cerebral Performance Category (CPC) score of 3 or 4 before OHCA. All enrolled patients received post-cardiac arrest care in accordance with current guidelines. Patients were closely monitored in the emergency intensive care unit, and TTM at 33 °C was introduced for comatose patients, regardless of the initial cardiac rhythm. Multimodal neurological prognosis was evaluated 72 h after either cardiac arrest. Patients who exhibited an active infection (as confirmed by the initial blood culture) immediately upon ROSC, a history of malignancy, and only one documented NLR or PLR value was excluded from the study. The study protocol was reviewed and approved by the Institutional Review Board of all participating hospitals. Informed consent was waived given the utilization of collected registry data (IRB No 2024-0482).

### 2.2. Data Collection and Outcomes

Data extracted from the web-based registry included information such as age, sex, comorbidities, cardiac arrest cause, initial documented rhythm, witnessed cardiac arrest, bystander CPR, blood culture results, lactate and laboratory findings during different phases of TTM (i.e., total white blood cell counts, neutrophil count, lymphocyte count, and platelet count). Prehospital down time was defined as the interval between the collapse and the arrival of the first professional medical responder. CPR duration was measured as the elapsed time from the initiation of the first professional chest compression until the first ROSC. Time to ROSC was calculated as the total time from the collapse to the first successful ROSC. The two indices were calculated based on the same blood samples: the NLR was determined by dividing the total neutrophil count by the total lymphocyte count, whereas the PLR was determined by dividing the platelet count by the total lymphocyte count. The NLR and PLR values were measured at three different phases: at 0, 24, and 48 h immediately after ROSC; at 24 h after ROSC; and at 48 h after ROSC. The primary outcome measured in this study was a poor neurological outcome, defined as a CPC score of 3–5 at 6 months.

### 2.3. Statistical Analysis

Continuous variables were presented as means with standard deviations (normal distribution) or medians with interquartile ranges, depending on data distribution. Categorical variables were expressed as numbers and percentages. The normal distribution of continuous variables was assessed using the Shapiro–Wilk test. Normally distributed data were analyzed using Student’s *t*-tests, whereas non-normally distributed data were analyzed using Mann–Whitney U tests. Chi-square or Fisher’s exact tests were performed for categorical variables.

We performed a Cox regression analysis to calculate hazard ratios (HR) and their 95% confidence intervals (CI) for predicting poor neurological outcomes. Variables with a p-value of less than 0.1 in the univariable analysis were included in the multivariable model. The cutoff values for the NLR and PLR, which offered the optimal balance of sensitivity and specificity, were determined using receiver operator (ROC) curve analysis with the Youden index to distinguish between outcomes. Statistical analysis was conducted using the SPSS 20.0 software package (SPSS Inc., Chicago, IL, USA).

## 3. Results

### 3.1. Baseline Characteristics

**Figure 1:**
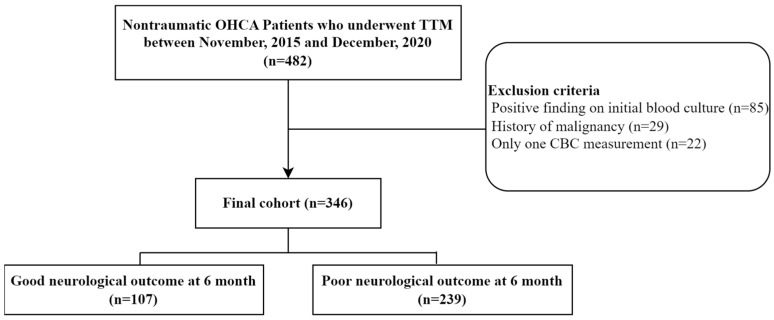
Patient flow diagram. OHCA, out-of-hospital cardiac arrest; TTM, targeted temperature management; CBC, complete blood count.

Among the 482 patients with OHCA treated with TTM, 136 were excluded due to positive findings on initial blood culture (85 cases), a history of malignancy (29 cases) and only one CBC measurement (22 cases). A total of 346 patients were finally included in the analysis. Among them, 107 (30.9%) and 239 (69.1%) patients showed good and poor neurological outcomes at 6 months, respectively (Figure 1).

Patients with good neurological outcomes were younger [52.0 (40.0–61.5) vs. 60.0 (49.0–73.0) years; *p* < 0.001], had a shorter CPR duration [17.0 (11.0–25.0) vs. 34.0 (22.0–46.0); *p* < 0.001], and a shorter total time to ROSC [28.5 (20.0–36.0) vs. 43.0 (30.0–58.0); *p* < 0.001]. Additionally, they had a higher incidence of witnessed cardiac arrest (79.4% vs. 56.5%; *p* < 0.001), an initial shockable rhythm (68.2% vs. 19.7%; *p* < 0.001), a presumed cardiac cause (85.0% vs. 47.3%; *p* < 0.001), and a history of acute coronary syndrome (23.4% vs. 13.4%; *p* = 0.021; Table 1).

### 3.2. Comparison of Early Post-ROSC Adverse Events and Cardiac Interventions

There were no statistically significant differences in adverse events during the first 7 days after ROSC, including significant bleeding requiring transfusion, infections requiring antibiotics, and re-arrest rates. However, cardiac interventions, specifically coronary angiography (CAG) and percutaneous coronary intervention (PCI), were significantly more common in the group with good neurological outcomes. On the other hand, the rate of extracorporeal membrane oxygenation (ECMO) did not differ significantly between the groups (Table 2).

### 3.3. Trends in the NLR and PLR During the First 48 h After ROSC

**Figure 2:**
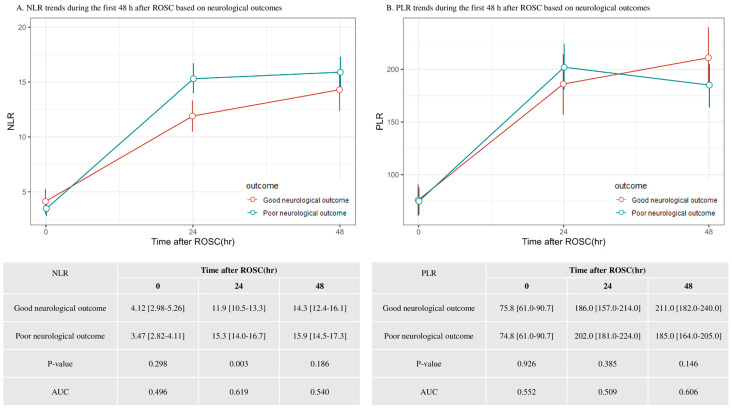
The trends in the neutrophil-to-lymphocyte ratio (NLR) and platelet-to-lymphocyte ratio (PLR) during the first 48 h after the return of spontaneous circulation (ROSC) based on neurologic outcomes.

The patients exhibited an overall increase in the NLR over time, with a significant increase within the first 24 h, followed by a comparatively modest increase between 24 and 48 h. However, an increase in the PLR was observed within the first 24 h, followed by a decline among those with poor neurological outcomes between 24 and 48 h, but a simultaneous increase among those with good neurological outcomes. The NLR at 24 h after ROSC was significantly correlated with poor neurological outcomes [11.9 (10.5–13.3) vs. 15.3 (14.0–16.7); *p* = 0.003], but no such correlation was observed for the NLR or PLR measured at other timepoints throughout post-cardiac arrest care (Figure 2). The NLR at 24 h after ROSC had an area under the ROC curve of 0.619 for predicting poor neurological outcomes at 6 months.

### 3.4. Factors Associated with Poor Neurologic Outcomes

In the univariable analysis, the NLR at 24 h after ROSC was significantly associated with poor neurological outcomes (HR: 1.02; 95% CI: 1.01–1.03; *p* = 0.002). After adjusting for statistically significant covariates identified in the univariable analysis, the NLR at 24 h after ROSC remained significantly associated with poor neurological outcomes (HR: 1.01; 95% CI: 1.00–1.03; *p* = 0.045; Table 3).

ROC curve analysis, utilizing the Youden index, identified that an NLR of 9 at 24 h after ROSC was the optimal cutoff for predicting poor neurological outcomes at 6 months. This threshold yielded a sensitivity of 72.5% and specificity of 47.7% (Table 4). The cutoff values needed to achieve 99% sensitivity and 99% specificity were determined to be 1.07 and 34.6, respectively.

## 4. Discussion

This study evaluated the association between the NLR and PLR at 0, 24, and 48 h after ROSC and poor neurological outcomes at 6 months in OHCA patients receiving TTM. Our findings show that the NLR at 24 h, but not the PLR, was an early predictor of poor neurologic outcomes (HR: 1.01; 95% CI: 1.00–1.03; *p* = 0.045).

Whole-body ischemic–reperfusion injury after ROSC can contribute to adverse outcomes. This process triggers a substantial surge in pulmonary blood flow, swift reoxygenation of ischemic tissue, and an increase in the production of reactive oxygen species [18]. Ischemic–reperfusion injury triggers inflammation through various pathways, including neutrophil activation, platelet activation and exhaustion, and the release of inflammatory cytokines [19]. Consequently, changes in blood cell counts and ratios during the acute phase following ROSC may be used to predict mortality and neurological outcomes [11]. Endogenous catecholamine and cortisol are released in response to physiological stress, causing an increase in neutrophils and a decrease in lymphocytes [20]. Additionally, lymphocyte apoptosis occurs in sepsis, which causes lymphopenia and an elevated NLR. Our results indicate that dynamic alterations in the NLR during the early stages after ROSC (i.e., at 24 h) were the only indices associated with poor outcomes. This result is supported by studies on sepsis, which have shown that this response promptly occurs within 4 to 8 h of an acute insult, making the NLR a superior indicator of acute illness compared to leukocytosis [21].

However, Weiser et al. reported in 2017 that the NLR measured upon hospital admission was associated with mortality independent of epinephrine administration. The aforementioned study was the first to evaluate the ability of the NLR to predict mortality in patients with OHCA and suggested that those with an NLR of ≥6 had a lower survival rate than did those with an NLR of <6 (hazard ratio: 1.52 [1.03–2.24]) [17]. This finding contradicts the results presented in the current study, which showed that only NLR at 24 h was associated poor neurological outcomes. One possible explanation for this discrepancy is that our study only included OHCA survivors receiving TTM. This is significant considering that neutrophils are inhibited during the hypothermic period and begin to increase after body temperature is normalized. Additionally, our study excluded patients with infections, such as bacteremia and pneumonia, unlike the aforementioned study. Variations in the characteristics of the cohorts, such as a higher proportion of patients with a non-shockable rhythm and the lower proportion of those with witnessed cardiac arrests and a presumed cardiac cause in our cohort, could have also contributed to the differing results. This is supported by a study including OHCA patients with a presumed cardiac cause, which showed that the optimal cutoff of the NLR for survival is 2.6 [22].

We successively measured the NLR up to 48 h, which provided more information about the NLR dynamics between hypothermia and normothermia. OHCA survivors in the current study exhibited an increase in their NLR from the time of admission to hypothermia (at 24 h). Patients with poor neurological outcomes had a higher NLR at 24 h after ROSC than did those with good neurological outcomes (15.3 vs. 11.9, respectively). This finding was consistent with a small single-center study (*n* = 95) that showed no significant difference in the NLR on admission and during rewarming between survivors and decedents. During cooling, however, decedents had a significantly higher NLR than did survivors [12]. The lack of association between the NLR at 48 and 78 h after ROSC and poor neurological outcomes observed in our study may imply that TTM interventions during this post-ROSC period may influence the inflammatory response.

The PLR has been increasingly recognized as an indicator of the inflammatory process and has been shown to have good prognostic value in patients with cancers and acute myocardial infarction [23,24,25]. One study showed that, among patients who suffered an in-hospital cardiac arrest, those with a PLR of over 180 exhibited approximately a200% increase in the risk of 30-day mortality, suggesting that the PLR could be used as an indicator of short-term mortality [26]. However, our study was unable to demonstrate such a correlation between PLR and hospital mortality. This discrepancy may be linked to the time course of platelet counts in patients undergoing TTM after OHCA [27]. A recent study that evaluated the systemic immune–inflammatory index as a prognostic marker of OHCA showed that the PLR was not associated with survival to discharge, which supports our results that the PLR was not associated with outcomes.

Our results showed that NLR predicts prognosis, whereas PLR does not, potentially reflecting coagulofibrinolytic changes in post-cardiac arrest syndrome (PCAS). Patients with PCAS experience distinct coagulofibrinolytic alterations, including heightened coagulation and impaired fibrinolysis [28]. The no-reflow phenomenon, first reported by Ames et al., is characterized by impaired reperfusion following cerebral ischemia, despite stable systemic circulation [29]. These findings suggest that coagulofibrinolytic changes in PCAS patients play a significant role in post-cardiac arrest brain injury. Platelets contribute to this phenomenon through tissue-factor-initiated coagulation, as well as through platelet aggregation and activation, primarily resulting in a reduction in platelet count rather than alterations in platelet function [30,31,32]. Therefore, PLR was not associated with neurological outcomes.

Only a limited number of studies have proposed a potential association between the NLR and PLR, which serve as indicators of systemic inflammation, and the outcomes of patients after cardiac arrest [11,12,15]. However, these studies were limited by their small cohort sizes, single-center designs, and the failure to exclude patients with bacterial infections immediately after ROSC. Consequently, these studies may not accurately reflect the impact of ischemic–reperfusion injury alone. In contrast, our study was designed to overcome these limitations, thereby enhancing reliability of our findings. Consistent with previous studies, our findings regarding the NLR within 24 h after ROSC show that this biomarker could potentially serve as a tool for early neuro-prognostication in a multimodal approach for post-cardiac arrest patients considering its cost-effectiveness, rapid processing, and wide availability.

Our study has several limitations. Firstly, this was a retrospective observational study with a relatively small cohort despite including multiple centers. Secondly, given that the NLR and PLR are inflammatory markers, confounding factors, such as early-phase infections, could have been present, although we did exclude certain conditions that could affect the NLR and PLR. Thirdly, despite adjusting for multiple confounding factors, some potential confounders could still have been overlooked in this study. Fourth, the inclusion of patients treated with TTM may have introduced individuals who experienced a relatively severe initial insult of cardiac arrest. This inclusion could have potentially confounded our results. Lastly, although each hospital followed the current guidelines for post-cardiac arrest care, variations may exist, potentially influencing our results.

## 5. Conclusions

We demonstrated that OHCA survivors exhibited an increase in the NLR during hypothermia period and that such an increase was significantly higher among patients with poor outcomes. In summary, the current study showed that the NLR at 24 h after ROSC, but not PLR, could potential be an early predictor of neurological outcomes in patients treated with TTM. Its cost-effectiveness, rapid availability, and simplicity make it an advantageous index for early neuro-prognostication in a multimodal approach.

## Figures and Tables

**Table 1:** Baseline characteristics of the study population based on the neurological outcomes.

	Total (*n* = 346)	Good Neurological Outcomes (*n* = 107)	Poor Neurological Outcomes (*n* = 239)	*p*-Value
Age	58.0 [46.0–69.8]	52.0 [40.0–61.5]	60.0 [49.0–73.0]	<0.001
Sex (male)	242 (69.9%)	82 (76.6%)	160 (66.9%)	0.069
**Medical history**				
Hypertension	119 (34.4%)	33 (30.8%)	86 (36.0%)	0.352
DM	81 (23.4%)	15 (14.0%)	66 (27.6%)	0.006
Acute coronary syndrome	57 (16.5%)	25 (23.4%)	37 (13.4%)	0.021
Arrhythmia	18 (5.2%)	7 (6.5%)	11 (4.6%)	0.453
Heart failure	11 (3.2%)	14 (3.7%)	7 (2.9%)	0.948
Cerebrovascular disease	35 (10.1%)	5 (4.7%)	30 (12.6%)	0.040
Chronic renal disease	32 (9.2%)	4 (3.7%)	28 (11.7%)	0.030
**Arrest characteristics**				
Witnessed arrest	220 (63.6%)	85 (79.4%)	135 (56.5%)	<0.001
Bystander CPR	207 (59.8%)	72 (67.3%)	135 (56.5%)	0.058
Initial shockable rhythm	120 (34.7%)	73 (68.2%)	47 (19.7%)	<0.001
Presumed cardiac cause	204 (59.0%)	91 (85.0%)	113 (47.3%)	<0.001
Prehospital down time, min	7.0 [4.5–12.0]	7.0 [5.0–11.0]	7.0 [4.0–12.0]	0.190
CPR duration, min	29.0 [17.0–41.8]	17.0 [11.0–25.0]	34.0 [22.0–46.0]	<0.001
Total time to ROSC, min	38.0 [23.0–55.0]	28.5 [20.0–36.0]	43.0 [30.0–58.0]	<0.001
**Vital signs**				
Systolic pressure, mmHg	120.0 [90.0–156.0]	120.0 [100.0–160.0]	120.0 [86.0–156.0]	0.190
Diastolic pressure, mmHg	70.0 [53.0–90.0]	78.0 [50.0–89.8]	69.5 [50.0–89.8]	0.011
Pulse rate, beats/min	105.3 ± 29.7	101.2 ± 28.1	107.0 ± 30.3	0.103
**Laboratory findings,** **Initial**				
White blood cell, 10^3^/µL	12.8 [9.7–17.5]	13.4 [10.0–17.3]	12.4 [9.15–17.6]	0.392
Platelet, 10^3^/µL	184 [145–234]	190 [156–247]	180 [156–228]	0.095
Total bilirubin, mg/dL	0.6 [0.4–0.8]	0.6 [0.4–0.9]	0.5 [0.3–0.8]	0.009
Creatinine, mg/dL	1.30 [1.10–1.70]	1.20 [1.00–1.10]	1.35 [1.40–1.90]	<0.001
Lactate, mmol/L	10.4 [6.3–13.5]	6.9 [4.5–11.0]	11.2 [8.0–14.1]	<0.001
Troponin I, ng/dL	0.06 [0.01–0.30]	0.06 [0.01–0.20]	0.06 [0.01–0.40]	0.998
CRP, mg/dL	0.14 [0.07–0.60]	0.12 [0.06–0.50]	0.17 [0.07–0.90]	0.179

Values are presented as a median (interquartile range) or number (percentage). DM, diabetes mellitus; CPR, cardiopulmonary resuscitation.

**Table 2:** Comparison of adverse events and interventions during the 7 days after ROSC.

	Total (*n* = 346)	Good Neurological Outcomes (*n* = 107)	Poor Neurological Outcomes (*n* = 239)	*p*-Value
**Adverse events**				
Significant bleeding requiring transfusion	15 (4.3%)	5 (4.7%)	10 (4.2%)	1.000
Infection requiring antibiotics	167 (48.3%)	57 (53.3%)	110 (46.0%)	0.258
Re-arrest	36 (10.4%)	6 (5.6%)	30 (12.6%)	0.057
**Intervention**				
Extracorporeal membrane oxygenation	25 (7.2%)	10 (9.3%)	15 (6.3%)	0.427
Coronary angiography	148 (42.8%)	75 (70.1%)	73 (30.5%)	<0.001
Percutaneous coronary intervention	64 (18.5%)	33 (30.8%)	31 (13.0%)	<0.001

**Table 3:** Univariable and multivariable analysis for predicting poor neurological outcome at 6 months.

	Univariable Analysis			Multivariable Analysis		
	HR	95% CI	*p*-Value	HR	95% CI	*p*-Value
NLR at 24 h	1.02	1.01–1.03	0.002	1.01	1.00–1.03	0.045
Age	1.02	1.01–1.02	<0.001	1.01	1.01–1.02	0.001
Sex (female)	1.27	0.96–1.66	0.092	1.12	0.84–1.48	0.442
Unwitnessed cardiac arrest	1.67	1.28–2.17	<0.001	0.98	0.74–1.32	0.918
Non-shockable rhythm	3.66	2.61–5.14	<0.001	2.70	1.81–4.02	<0.001
Bystander CPR	1.34	1.03–1.74	0.027	0.95	0.72–1.25	0.694
Presumed cardiac cause	2.36	1.82–3.07	<0.001	1.24	0.91–1.70	0.177
Total time to ROSC	1.02	1.01–1.02	<0.001	1.01	1.01–1.02	<0.001
Lactate	1.05	1.03–1.07	<0.001	1.05	1.02–1.07	<0.001

Abbreviations: HR, hazard ratios; CI, confidence intervals; NLR, neutrophil-to-lymphocyte ratio; CPR, cardiopulmonary resuscitation; ROSC, return of spontaneous circulation.

**Table 4:** Optimal cutoff values calculated using the Youden Index.

	Cut Off	Sens	Spec	PPV	NPV
NLR at 24 h	9	72.5%	47.7%	75%	44.4%
	1.07	99.2%	0.1%	68.8%	0%
	34.6	0.1%	99.1%	92.3%	31.7%

Abbreviation: NLR, neutrophil-to-lymphocyte ratio; Sens, sensitivity; Spec, specificity; PPV, positive predictive value; NPV, negative predictive value.

## Data Availability

The datasets are available from corresponding authors upon reasonable request.

## References

[1] Panchal A.R., Bartos J.A., Cabañas J.G., Donnino M.W., Drennan I.R., Hirsch K.G., Kudenchuk P.J., Kurz M.C., Lavonas E.J., Morley P.T. (2020). Part 3: Adult Basic and Advanced Life Support: 2020 American Heart Association Guidelines for Cardiopulmonary Resuscitation and Emergency Cardiovascular Care. Circulation.

[2] Kim Y.-M., Jeung K.W., Kim W.Y., Park Y.S., Oh J.S., You Y.H., Lee D.H., Chae M.K., Jeong Y.J., Kim M.C. (2021). 2020 Korean Guidelines for Cardiopulmonary Resuscitation. Part 5. Post-Cardiac Arrest Care. Clin. Exp. Emerg. Med..

[3] Laver S., Farrow C., Turner D., Nolan J. (2004). Mode of Death after Admission to an Intensive Care Unit Following Cardiac Arrest. Intensive Care Med..

[4] Dufner M.C., Andre F., Stiepak J., Zelniker T., Chorianopoulos E., Preusch M., Katus H.A., Leuschner F. (2016). Therapeutic Hypothermia Impacts Leukocyte Kinetics after Cardiac Arrest. Cardiovasc. Diagn. Ther..

[5] Cotoia A., Franchi F., De Fazio C., Vincent J.-L., Creteur J., Taccone F.S. (2018). Platelet Indices and Outcome after Cardiac Arrest. BMC Emerg. Med..

[6] Woo S.H., Lee W.J., Kim D.H., Cho Y., Cho G.C. (2020). Initial Red Cell Distribution Width as a Predictor of Poor Neurological Outcomes in Out-of-Hospital Cardiac Arrest Survivors in a Prospective, Multicenter Observational Study (the KoCARC Study). Sci. Rep..

[7] Cao C., Yu M., Chai Y. (2019). Pathological Alteration and Therapeutic Implications of Sepsis-Induced Immune Cell Apoptosis. Cell Death Dis..

[8] Li S., Hu L., Wang J., Zou F., Han B., Wang Y., Liu K. (2022). Prolonged Increased Neutrophil-to-Lymphocyte Ratio Is Associated with Mortality after Successful Revascularization for Treatment of Acute Ischemic Stroke. BMC Neurol..

[9] Fest J., Ruiter T.R., Groot Koerkamp B., Rizopoulos D., Ikram M.A., van Eijck C.H.J., Stricker B.H. (2019). The Neutrophil-to-Lymphocyte Ratio Is Associated with Mortality in the General Population: The Rotterdam Study. Eur. J. Epidemiol..

[10] White B.C., Grossman L.I., O’Neil B.J., DeGracia D.J., Neumar R.W., Rafols J.A., Krause G.S. (1996). Global Brain Ischemia and Reperfusion. Ann. Emerg. Med..

[11] Huang Y.-H., Lin Y.-S., Wu C.-H., How C.-K., Chen C.-T. (2023). Prognostic Value of Neutrophil-Lymphocyte Ratio in out-of-Hospital Cardiac Arrest Patients Receiving Targeted Temperature Management: An Observational Cohort Study. J. Formos. Med. Assoc..

[12] Başer K. (2017). Changes in Neutrophil-to-Lymphocyte Ratios in Postcardiac Arrest Patients Treated with Targeted Temperature Management. Anatol. J. Cardiol..

[13] Tanrikulu C.S. (2016). Neutrophil-to-Lymphocyte Ratio and Platelet-to-Lymphocyte Ratio in Cardiac and Non-Cardiac Arrest Distinction: A Retrospective Cohort Study. Acta Medica.

[14] Huang L., Peng J., Wang X., Li F. (2021). High Platelet-Lymphocyte Ratio Is a Risk Factor for 30-Day Mortality In In-Hospital Cardiac Arrest Patients: A Case-Control Study. Expert Rev. Clin. Immunol..

[15] Kim H.J., Park K.N., Kim S.H., Lee B.K., Oh S.H., Moon H.K., Jeung K.W., Choi S.P., Cho I.S., Youn C.S. (2018). Association between the Neutrophil-to-Lymphocyte Ratio and Neurological Outcomes in Patients Undergoing Targeted Temperature Management after Cardiac Arrest. J. Crit. Care.

[16] Wang D., Yang J.-X., Cao D.-Y., Wan X.-R., Feng F.-Z., Huang H.-F., Shen K., Xiang Y. (2013). Preoperative Neutrophil-Lymphocyte and Platelet-Lymphocyte Ratios as Independent Predictors of Cervical Stromal Involvement in Surgically Treated Endometrioid Adenocarcinoma. Onco Targets Ther..

[17] Weiser C., Schwameis M., Sterz F., Herkner H., Lang I.M., Schwarzinger I., Spiel A.O. (2017). Mortality in Patients Resuscitated from Out-of-Hospital Cardiac Arrest Based on Automated Blood Cell Count and Neutrophil Lymphocyte Ratio at Admission. Resuscitation.

[18] Yokoyama H., Nagao K., Hase M., Tahara Y., Hazui H., Arimoto H., Kashiwase K., Sawano H., Yasuga Y., Kuroda Y. (2011). Impact of Therapeutic Hypothermia in the Treatment of Patients with Out-of-Hospital Cardiac Arrest From the J-PULSE-HYPO Study Registry. Circ. J..

[19] Gando S., Wada T. (2019). Disseminated Intravascular Coagulation in Cardiac Arrest and Resuscitation. J. Thromb. Haemost..

[20] Onsrud M., Thorsby E. (1981). Influence of in Vivo Hydrocortisone on Some Human Blood Lymphocyte Subpopulations. Scand. J. Immunol..

[21] Zhang Y., Li J., Lou J., Zhou Y., Bo L., Zhu J., Zhu K., Wan X., Cai Z., Deng X. (2011). Upregulation of Programmed Death-1 on T Cells and Programmed Death Ligand-1 on Monocytes in Septic Shock Patients. Crit. Care.

[22] Taha Sert E., Kokulu K., Mutlu H., Gül M., Uslu Y. (2023). Performance of the Systemic Immune-Inflammation Index in Predicting Survival to Discharge in out-of-Hospital Cardiac Arrest. Resusc. Plus.

[23] Balta S., Ozturk C. (2015). The Platelet-Lymphocyte Ratio: A Simple, Inexpensive and Rapid Prognostic Marker for Cardiovascular Events. Platelets.

[24] Zhou X., Du Y., Huang Z., Xu J., Qiu T., Wang J., Wang T., Zhu W., Liu P. (2014). Prognostic Value of PLR in Various Cancers: A Meta-Analysis. PLoS ONE.

[25] Kim Y.J., Kang J., Ryoo S.M., Ahn S., Huh J.W., Kim W.Y. (2019). Platelet-lymphocyte ratio after granulocyte colony stimulating factor administration: An early prognostic marker in septic shock patients with chemotherapy-induced febrile neutropenia. Shock.

[26] Seo M., Yamada T., Morita T., Furukawa Y., Tamaki S., Iwasaki Y., Kawasaki M., Kikuchi A., Kawai T., Ikeda I. (2018). P589 Prognostic Value of Systemic Immune-Inflammation Index in Patients with Chronic Heart Failure. Eur. Heart J..

[27] Kim H.J., Park K.N., Kim S.H., Lee B.K., Oh S.H., Jeung K.W., Cho I.S., Youn C.S. (2019). Time Course of Platelet Counts in Relation to the Neurologic Outcome in Patients Undergoing Targeted Temperature Management after Cardiac Arrest. Resuscitation.

[28] Böttiger B.W., Motsch J., Böhrer H., Böker T., Aulmann M., Nawroth P.P., Martin E. (1995). Activation of Blood Coagulation After Cardiac Arrest Is Not Balanced Adequately by Activation of Endogenous Fibrinolysis. Circulation.

[29] Ames A., Wright R.L., Kowada M., Thurston J.M., Majno G. (1968). Cerebral Ischemia. II. The No-Reflow Phenomenon. Am. J. Pathol..

[30] Wada T. (2017). Coagulofibrinolytic Changes in Patients with Post-Cardiac Arrest Syndrome. Front. Med..

[31] Nolan J.P., Neumar R.W., Adrie C., Aibiki M., Berg R.A., Böttiger B.W., Callaway C., Clark R.S.B., Geocadin R.G., Jauch E.C. (2008). Post-Cardiac Arrest Syndrome: Epidemiology, Pathophysiology, Treatment, and Prognostication. A Scientific Statement from the International Liaison Committee on Resuscitation; the American Heart Association Emergency Cardiovascular Care Committee; the Council on Cardiovascular Surgery and Anesthesia; the Council on Cardiopulmonary, Perioperative, and Critical Care; the Council on Clinical Cardiology; the Council on Stroke. Resuscitation.

[32] Roberts B.W., Kilgannon J.H., Chansky M.E., Mittal N., Wooden J., Parrillo J.E., Trzeciak S. (2013). Multiple Organ Dysfunction after Return of Spontaneous Circulation in Postcardiac Arrest Syndrome. Crit. Care Med..

